# Non-small cell lung cancer and metabolism research from 2013 to 2023: a visual analysis and bibliometric study

**DOI:** 10.3389/fonc.2024.1322090

**Published:** 2024-05-28

**Authors:** Jin Yang, Wei Yang, Jie Zhang, Aiping Huang, Shiyuan Yin, Hua Zhang, Zongrui Luo, Xiaojuan Li, Yihua Chen, Lijie Ma, Chao Wang

**Affiliations:** ^1^ Department of Pathology, Affiliated Hospital of North Sichuan Medical College, Nanchong, China; ^2^ Department of Pathology, General Hospital of Western Theater Command, Chengdu, China; ^3^ Affiliated Hospital of Southwest Jiaotong University, General Hospital of Western Theater Command, Chengdu, China; ^4^ Department of Pulmonary and Critical Care Medicine, General Hospital of Western Theater Command, Chengdu, China; ^5^ Department of Library, Chengdu University of Traditional Chinese Medicine, Chengdu, China; ^6^ Department of Pathology, Affiliated Hospital of Southwest Medical University, Luzhou, China; ^7^ Department of Human Resource, Yibin Sixth People’s Hospital, Yibin, China

**Keywords:** non-small cell lung cancer, NSCLC, metabolism, bibliometric, immunotherapy, checkpoint inhibitor

## Abstract

**Background:**

As one of the most prevalent primary lung tumors, non-small cell lung cancer (NSCLC) has garnered considerable research interest due to its high metastasis rates and poor prognosis outcomes. Across different cancer types, metabolic processes are required for tumors progression and growth, thus interfering with such processes in NSCLC may therapeutically viable for limiting/halting disease progression. Therefore, comprehending how metabolic processes contribute to growth and survival mechanisms in cancers, including NSCLC, may elucidate key functions underpinning tumor cell metabolism. However, no bibliometric analyses have been published in this field, therefore we address this knowledge gap here.

**Methods:**

Between 2013 and 2023 (December 28^th^), articles related to the NSCLC and metabolism (NSCLC-Met) field were retrieved from the Web of Science Core Collection (WoSCC). To fully dissect NSCLC-Met research directions and articles, we used the Bibliometrix package in R, VOSviewer and CiteSpace software to visually represent global trends and hotspots.

**Results:**

Between 2013 and 2023, 2,246 NSCLC-Met articles were retrieved, with a continuous upward trend and rapid development observed year on year. *Cancers* published the most articles, with *Cancer Research* recording the highest average citation numbers. Zhang Li from China was the most prolific author, but the highest number of authors came from the USA. China, USA, and Italy were the top three countries with the highest number of published articles, with close cooperation identified between countries. Recent hotspots and research directions were reflected by “lung adenocarcinoma”, “immunotherapy”, “nivolumab”, “checkpoint inhibitors”, “blockade”, and “pembrolizumab”, while “gut microbiome”, “egfr” and “dose painting” were important topics for researchers.

**Conclusion:**

From our analyses, scientists can now explore new hotspots and research directions in the NSCLC-Met field. Further in-depth research in this field will undoubtedly provide more new insights on disease diagnostics, treatment, and prognostics.

## Introduction

Globally, lung cancer (LC) is responsible for the highest number of cancer-related deaths (1, 2), with cancer statistics showing that the 5-year survival rate for the disease was 19% in 2020 (3). Non-small cell lung cancer (NSCLC) accounts for approximately 85% of LC histological types, which mainly include squamous cell carcinoma and adenocarcinoma (4). In China, LC is the main cause of cancer-related morbidity and mortality, therefore, this scenario represents unique circumstances and specific challenges given China’s large population, changing demographics, and unique disease treatment methods (5). However, as advanced LC incidences decrease with improved access to early screening and treatments, the incidence of locally staged LC has increased considerably by 4.5%/year. As a consequence, the proportion of locally staged diagnoses has increased from 17% in 2004 to 28% in 2018, while 3-year relative survival rates have increased from 21% to 31% (2). Several methods are used to treat NSCLC; while surgery remains the favored treatment (6), cytotoxic chemotherapy is a viable systemic therapy for most patients. Similarly, molecular targeted therapies or immunotherapies represent first-line standard treatment options for approximately half of patients with advanced NSCLC, but their long-term survival is poor (7).

By identifying the “Warburg effect”, Otto Warburg posited that metabolic differences existed between normal tissues and tumors. Metabolic reprogramming is a critical process in tumors (8), including LC, and involves lipid, amino acid, glucose, nucleotide, mitochondrial oxidative metabolism, and autophagy processes (9). Lipid metabolism has been extensively studied in recent years, with lipid production, uptake, and storage mechanism all upregulated in NSCLC, thereby promoting rapid tumor cell growth and proliferation (10). Therefore, the in-depth characterization of NSCLC metabolic profiles may facilitate individualized therapies and improve prognosis outcomes in patients, and provide new clinical treatment avenues for LC. In recent years, interest in the NSCLC and metabolism (NSCLC-Met) field has increased considerably; scientists, using basic studies and clinical trials, have investigated if cancer development/progression may be halted by interfering with metabolic changes in different cancers, including NSCLC etiology (11, 12).

In our big data world, bibliometrics plays important roles in both practical and theoretical research, as bibliometric methods allow scientists to rapidly identify specific literature characteristics, comprehend development processes, identify research hotspots (13), predict research trends, and improve research outcomes (14). Bibliometric analyses can be used to visualize the impact of academic outputs and estimate their scientific relevance across many disciplines. Despite some methodological limitations, the approach is used to visually quantify academic achievements and their impact in many fields. To date, several bibliometric studies related to LC have been published, mainly on immunotherapy (15, 16), radiotherapy (17), gut microbiota (18), postoperative analgesia (19), anti-programmed death-1/programmed death-ligand 1 (anti-PD-1/PD-L1) therapy (20), machine learning in NSCLC radiotherapy (21), and chronic obstructive pulmonary disease (22). However, no bibliometric studies have been published in the NSCLC-Met field.

Here, we retrieved articles from the Web of Science Core Collection (WoSCC), and the Bibliometrix packages (based on R), VOSviewer and CiteSpace software were used to analyze numbers of articles, authors, countries/regions, institutions, references, journals, and keywords in the NSCLC-Met field from this work, scientists can better understand research hot spots, new directions, and future development trends in the NSCLC-Met field.

## Materials and methods

### Data sources and search strategy

From 2013 to 2023 (December 28^th^), the Science Citation Index Expanded database of the WoSCC (Thomson Scientific Information Group, USA) was used to perform article searches.

Search strategies included: TS = (“Carcinoma, Non-Small-Cell Lung” OR “Carcinoma, Non Small Cell Lung” OR “Carcinomas, Non-Small-Cell Lung” OR “Lung Carcinoma, Non-Small-Cell” OR “Lung Carcinomas, Non-Small-Cell” OR “Non-Small-Cell Lung Carcinomas” OR “Non-Small-Cell Lung Carcinoma” OR “Non Small Cell Lung Carcinoma” OR “Carcinoma, Non-Small Cell Lung” OR “Non-Small Cell Lung Carcinoma” OR “Non-Small Cell Lung Cancer” OR “Nonsmall Cell Lung Cancer”) AND TS = (“Metabolism” OR “Metabolic” OR “Metaboly “ OR “Metabolomics” OR “Anabolism” OR “Catabolism”).

### Inclusion and exclusion criteria

Searches were completed by two authors on December 28^th^ 2023 to limit bias caused by daily-database updates. The only language allowed in this study was English. The only documents available were articles and reviews. Two retracted articles were removed from the original data, and CiteSpace software was used to finalize literature deduplication and normalization.

### Statistical analyses

Articles were analyzed based on annual output, country, institution, journal, author, keywords, and citations, with a view to extracting characteristics and providing descriptive results. Bibliometrix packages based on R (v.4.3.1) were used for bibliometric analyses and data visualization; VOSviewer (v.1.6.19) was used to visualize networks related to authors, countries, and keywords. CiteSpace (v.6.2.R6) was used to construct correlation maps depicting country collaborations and keyword clustering. For burst detection, CiteSpace was also used to examine research directions and institutions with research potential. Microsoft Office Excel 2021 was used to cluster bar/line charts, and plot pie charts and tree maps.

## Results

### Annual article analyses

**Figure 1:**
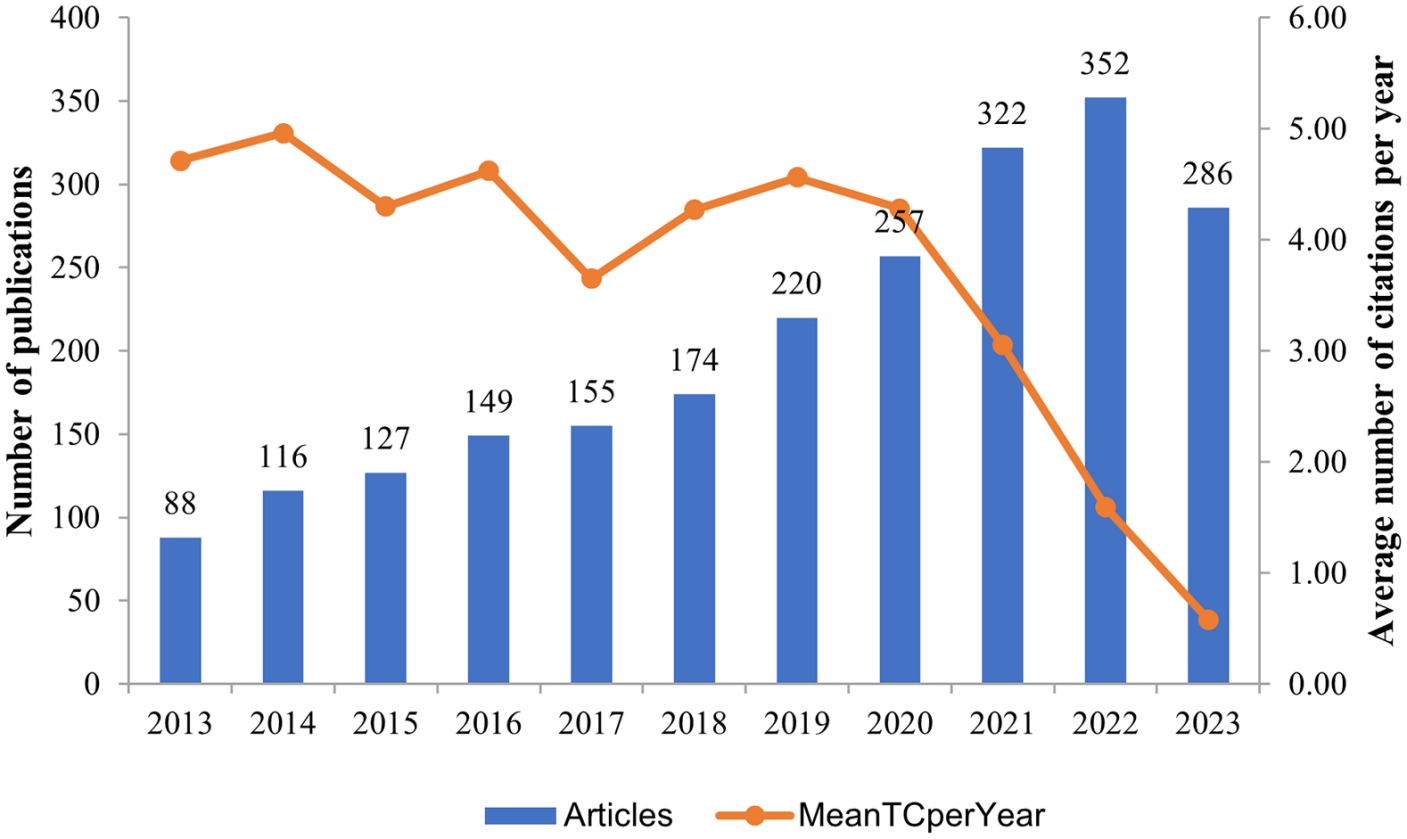
Annual article and average citation numbers (2013–2023) for non-small cell lung cancer and metabolism articles.

We included 2,246 articles. We separately counted article numbers/year and average citation numbers/year. In the previous decade, article numbers in the NSCLC-Met field have shown a continuous upward trend, with rapid development. Between 2013 and 2018, the net increase in article numbers/year ranged from 6 to 28 (809 in total), while between 2018 and 2022, the net increase in article numbers/year ranged from 30 to 65 (1,151 in total). Up to December 28^th^ 2023, 286 articles had been published in that year (**Figure 1**).

From 2013 to 2022, article numbers showed a steady upward trend and reflected gradual exploration in the NSCLC-Met field. In particular, 2022 had the highest numbers of articles, with 352 published, and suggested considerable research attention in this period. Additionally, the average number of citations/year was the highest in 2014 (4.96) (**Figure 1**).

### Journal analyses

In total, 606 journals published original articles on NSCLC-Met; the top ten most productive are shown (**Table 1**). In total, 417 articles were published in these ten journals. Of these, *Cancers* published the most articles (75), followed by *Frontiers in Oncology* (61), and *PLoS ONE* (50). In terms of citation numbers, *Cancer Research* had the highest number of citations (2528), followed by *Nature* (2216) and *Journal of Clinical Oncology* (2107). By examining productive–journal–distribution in the NSCLC-Met field, we provide a direction for article submission, especially for those articles with the highest citations, which in turn can help identify influential journals in the field, and timely track the latest literature and references for submission.

**Table 1:** Top ten productive journals in the non-small cell lung cancer and metabolism field.

Rank	Journals	Articles	IF(JCR2022)	JCR quartile	Rank	Cited Journal	Citations	IF(JCR2022)	JCR quartile
1	CANCERS	75	5.2	Q1	1	CANCER RES	2528	11.2	Q1
2	FRONT ONCOL	61	4.7	Q2	2	NATURE	2216	64.8	Q1
3	PLOS ONE	50	3.7	Q2	3	J CLIN ONCOL	2107	45.3	Q1
4	LUNG CANCER	46	5.3	Q1	4	CLIN CANCER RES	2075	11.5	Q1
5	ONCOL LETT	40	2.9	Q3	5	J THORAC ONCOL	1737	20.4	Q1
6	EUR J NUCL MED MOL I	39	9.1	Q1	6	CELL	1726	64.5	Q1
7	J NUCL MED	29	9.3	Q1	7	NEW ENGL J MED	1658	158.5	Q1
8	INT J MOL SCI	26	5.6	Q1	8	PLOS ONE	1553	3.7	Q1
9	CANCER RES	26	11.2	Q1	9	J NUCL MED	1551	9.3	Q2
10	THORAC CANCER	25	2.9	Q3	10	LUNG CANCER	1540	5.3	Q1

### Author analyses

**Figure 2:**
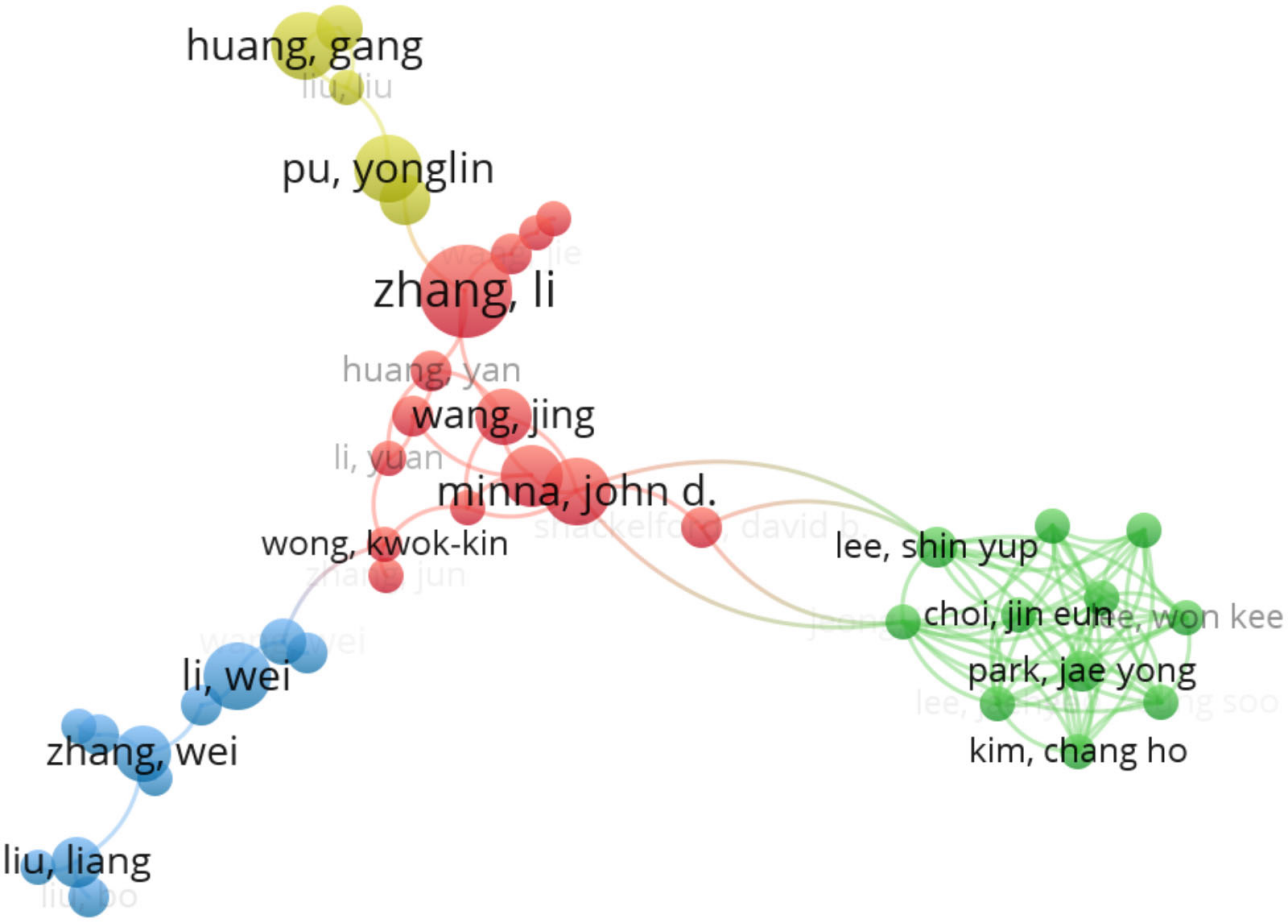
Co-authorship map. The size of the node reflects the number of papers published by the researcher, and the curves indicate collaborations between researchers. The nodes with the same color denote researchers who have collaborated on multiple projects or papers.

In total, 15,723 research scientists contributed to 2,246 original articles in the NSCLC-Met field. The top ten most active authors, with the most articles, are shown (**Table 2**). Zhang Li was the most prolific author with 18 articles, followed by Lopic Egesta (15), and Minna John D (13). The top ten authors were all cited > 90 times, with DeBerardinis Ralph being the most cited with 2,064 citations, followed by Minna John (1,474), and Guo Jessie Yanxiang (1,088). These authors were highly influential and important representatives in this field. A co-authorship map, which reflected the core authors in this field and indicated their cooperation and mutual citation relationships, was used to evaluate their academic influence in the field. Nodes represented authors, larger nodes indicated more published articles, while links represented author collaborations, and clusters were marked by different colors. Author collaboration analyses were performed in VOSviewer (**Figure 2**). Out of 15,723 authors, 222 had published at least five articles in the NSCLC-Met field.

**Table 2:** The top ten most prolific authors and cited authors in the non-small cell lung cancer and metabolism field.

Rank	Authors	Articles	Rank	Cited authors	Cite frequency
1	Zhang Li	18	1	DeBerardinis Ralph J	2064
2	Lopic Egesta	15	2	Minna John D	1474
3	Minna John D	13	3	Shawn Reuben J	1088
4	Choi Joon Young	13	4	Faubert Brandon	1079
5	Huang Gang	13	5	Kim Jiyeon	1066
6	Li Wei	13	6	Guo Jessie Yanxiang	973
7	Pu Yonglin	13	7	Shackelford David B	971
8	Castello Angelo	12	8	White Eileen	906
9	Kaira Kyoichi	12	9	Chen Pei-hsuan	807
10	DeBerardinis Ralph J	12	10	Jiang Lei	759

### Institution analyses

**Figure 3:**
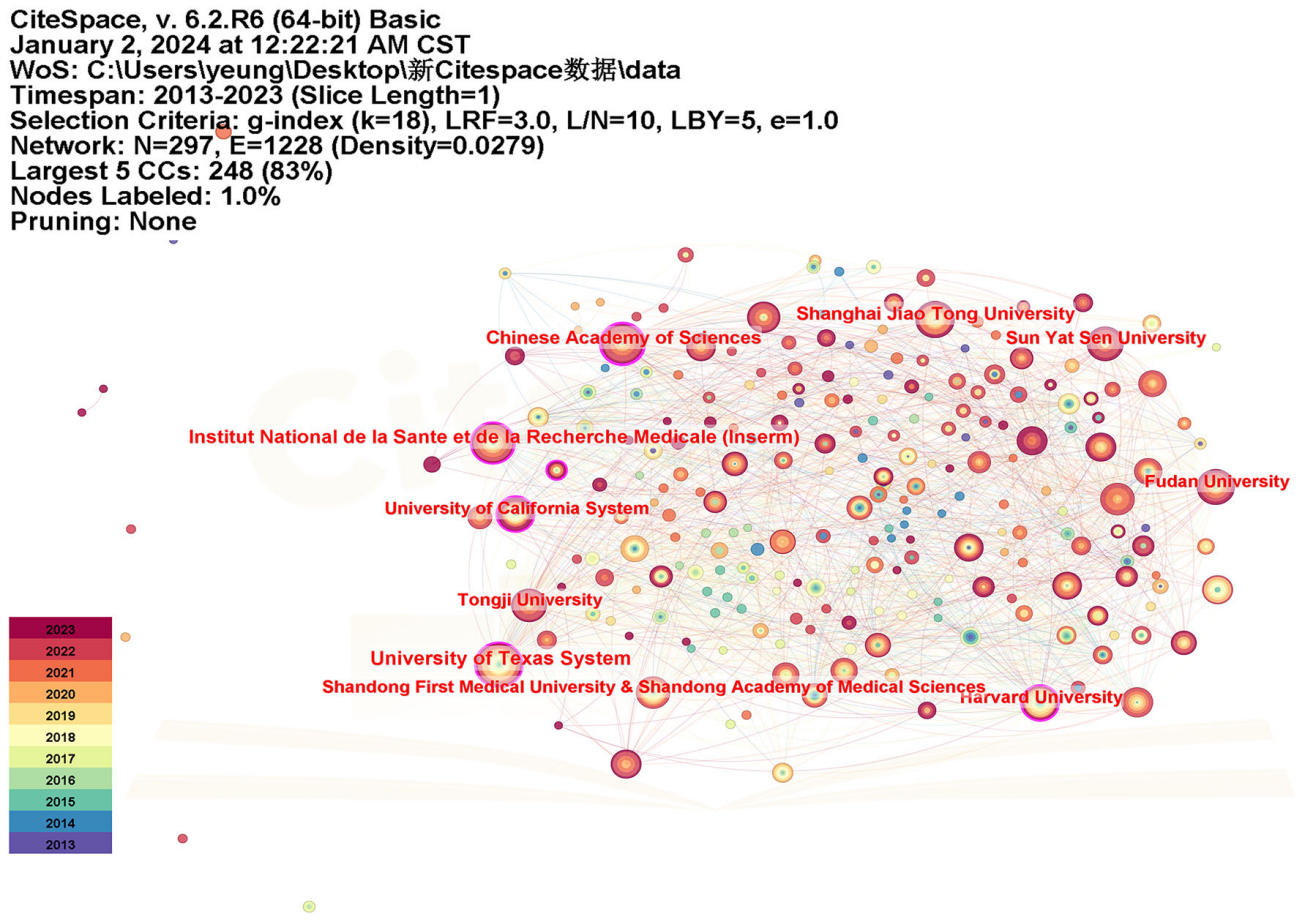
Institution collaboration map related to the non-small cell lung cancer and metabolism field. The node is represented in a concentric circle, and the node size reflects the number of articles published by the institution. Different colors of circles denote different years, while the width of each circle represents the number of articles published in the corresponding year. The purple circles outside some nodes reflect high betweenness centrality, indicating their importance as bridges in the network. The curves between nodes represent collaborative relationships among institution, with the thickness indicating the strength of connection and the color indicating the earliest year in which the connection was found.

To further understand institutional research contributions and their current status in the NSCLC-Met field, we counted articles published by different research institutions using CiteSpace. For parameter settings, we selected “institution” as node type, set the K value to a maximum of 18, and selected the 2013–2023 period. Considering data processing efficiency and accuracy, we divided the time into ten periods, with each period represented by 1 year, and analyzed institutional distribution data (**Table 3**, **Figure 3**).

**Table 3:** The top ten most productive institutions in terms of article numbers and intermediary centrality.

Rank	Institution	Counts	Year	Centrality
1	University of Texas System	77	2013	0.14
2	Institut National de la Sante et de la Recherche Medicale (Inserm)	61	2013	0.14
3	Shanghai Jiao Tong University	57	2014	0.04
4	Chinese Academy of Sciences	55	2014	0.22
5	Harvard University	47	2013	0.14
6	Sun Yat Sen University	46	2016	0.06
7	University of California System	44	2013	0.20
8	Fudan University	44	2016	0.08
9	Tongji University	42	2018	0.03
10	Shandong First Medical University & Shandong Academy of Medical Sciences	39	2013	0.03

We identified 297 institutions. The University of Texas System was relatively active; it was a scientific network center in the NSCLC-Met field, served as a bridge, and was one of the most widely published institutions. Published articles by research institutions were mainly concentrated in two regions: China and the USA. From a cooperative network perspective, the Chinese Academy of Sciences recorded the highest centrality; it had an extremely important position and influence, and had close cooperative relationships with other institutions.

### Country/region analyses

**Figure 4:**
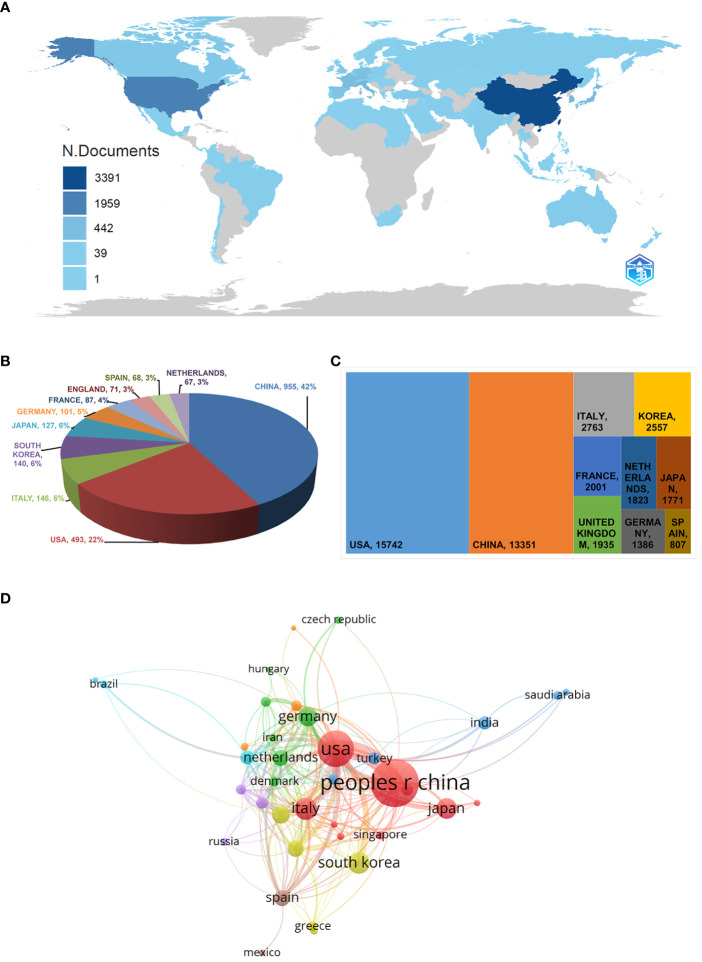
Country/region map related to non-small cell lung cancer and metabolism. **(A)** Geographical distribution of global output. **(B)** Pie chart showing the top ten countries/regions in terms of article numbers. **(C)** The top ten most cited countries/regions. **(D)** Visual cluster diagram showing cooperation between different countries. The node size reflects the country’s research activity levels, and the curves indicate collaborative relationships between different countries. The nodes with the same color denote countries who have similar scientific collaboration patterns. “people r china” here is referred to as “China” in the text.

From 2013 to 2023, 66 countries/regions conducted NSCLC-Met research; global article production is shown (**Figure 4A**). We used CiteSpace to select “country” as the node type and the K value was set to a maximum of 13. From the data, a pie chart was generated to show the top ten countries/regions with the highest article numbers (**Figure 4B**). Notably, in the past 10 years, China published the most NSCLC-Met articles (955), while the USA and Italy ranked two and three, respectively (**Figure 4B**). Among published articles, the USA had the highest citation numbers (15,472), followed by China (13,351), and Italy (2,763) (**Figure 4C**). We also used VOSviewer to generate a collaborative network including countries/regions with ≥ five articles. As shown (**Figure 4C**), close cooperation existed between countries; it was particularly noteworthy that China cooperated with almost all other countries, with a close cooperative relationship with the USA (**Figure 4D**).

### Keyword analyses

**Figure 5:**
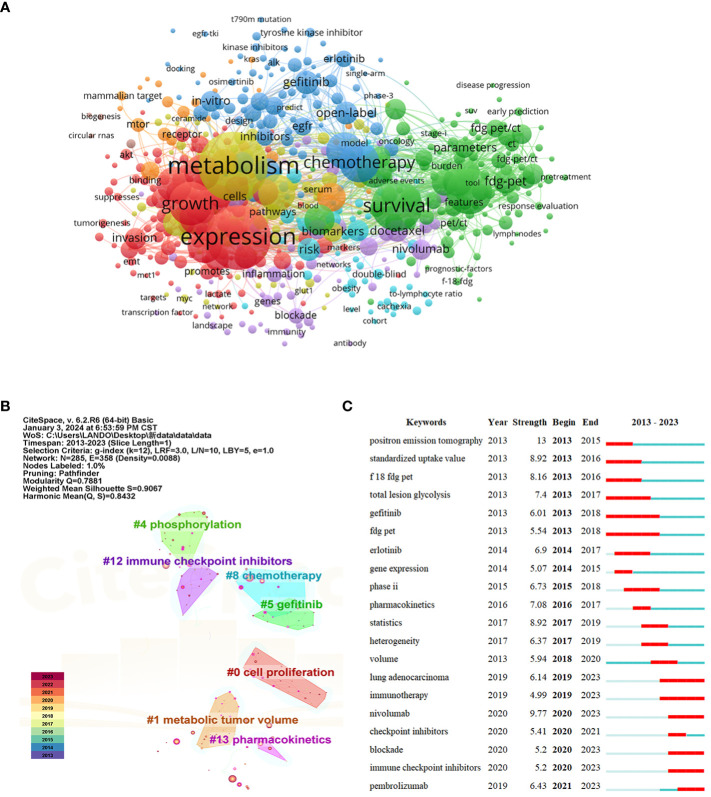
Keyword analyses related to the non-small cell lung cancer and metabolism (NSCLC-Met) field. **(A)** Network visualization of co-occurrence keywords. The node size reflects the keyword frequency of occurrence in the literature and the nodes with the same color display groups of keywords with similar research focus. **(B)** Keyword cluster map. The node size reflects the keyword frequency of occurrence in the literature, and the curves indicate the relevance between keywords. The nodes with the same color represent groups of keywords with similar research focus. **(C)** Top 20 highest-intensity keywords in the NSCLC-Met field. Each keyword burstiness is represented by the length of the line and the saturation of its color. Long and deeply colored lines indicate keywords with high burstiness, signifying a sharp increase in research interest in these keywords over a short period of time.

Keywords are highly refined terms found in core article themes and content. Based on extracted keywords, words with the same meaning, such as NSCLC, were removed and a network constructed in VOSviewer. Node size represented the frequency of keyword occurrence, connections between nodes represented interrelationships between keywords, and node color represented similarity in the main topic. As shown, cluster keywords were divided into four meaningful clusters (**Figure 5A**). We used CiteSpace to extract keywords from 2,246 articles, and node types were selected as “keywords” with a K value set to a maximum of 12. The top 20 keywords are shown (**Table 4**). Using CiteSpace for keyword clustering analyses, a keyword clustering map was generated with Q =0.7881 and S =0.9067. According to CiteSpace, NSCLC-Met studies were categorized into 13 major clusters. The cluster numbers are sorted by size, i.e. how much literature is included; clusters with smaller labels usually contain more keywords or nodes. We showed keyword clusters representing metabolic aspects of NSCLC research: “cell proliferation”, “metabolic tumor volume”, “phosphorylation”, “gefitinib”, “chemotherapy”, “immune checkpoint inhibitors”, and “pharmacokinetics” (**Figure 5B**).

**Table 4:** The top 20 occurrences keywords related to non-small cell lung cancer and metabolism.

Rank	Keywords	Occurrences	Rank	Keywords	Occurrences
1	non-small cell lung cancer	597	11	resistance	150
2	metabolism	477	12	therapy	148
3	expression	426	13	apoptosis	146
4	lung cancer	349	14	proliferation	144
5	survival	278	15	activation	140
6	positron emission tomography	260	16	inhibition	124
7	growth	213	17	prognostic value	113
8	chemotherapy	183	18	carcinoma	112
9	cancer	161	19	adenocarcinoma	102
10	cell lung cancer	160	20	pathway	90

Finally, from 2013 to 2023, we analyzed emerging vocabulary in the NSCLC-Met field. CiteSpace is highly advantageous as it can display sudden terminology bursts in a particular research field during a specific time period to potentially reflect hotspots and trends (23). Keywords appearing in earlier periods indicated that scientists were focusing on the field at earlier stages, while more current keywords indicated the topic had suddenly attracted attention (24). We used CiteSpace to analyze outbreak keywords in the NSCLC-Met field (**Figure 5C**); red lines after outbreak periods indicated a sudden keyword outbreak during that period. The main outbreak keywords in recent years were: “positron emission tomography”, “nivolumab”, “standardized uptake value”, “statistics” and “f 18 fdg pet”, with outbreak intensities of 13, 9.77, 8.92, 8.92, and 8.16, respectively. “Positron emission tomography”, “standardized uptake value”, “f 18 fdg pet”, “gefitinib”, and “total lesion glycolysis” keywords were the earliest outbreak keywords, while the most recent included “lung adenocarcinoma”, “immunotherapy”, “nivolumab”, “checkpoint inhibitors”, “blockade”, and “pembrolizumab”, which mainly appeared between 2019 and 2021. The keyword with the longest outbreak duration was “gefitinib” and “fdg pet”. Therefore, it was possible that NSCLC-Met research will revolve around these keywords in the coming several years.

### Cited reference analyses

By analyzing cited and co-cited references, we provided essential background information on the NSCLC-Met field. The top ten cited references (from 70,596 articles) are shown (**Table 5**), and indicated a high proportion of reviews. The most cited and co-cited articles were authored by Douglas Hanahan. In 2011, Douglas Hanahan and his colleague published the first edition of “Hallmarks of Cancer” in Cell, which has been cited more than 30,000 times. The six hallmarks of cancer are self-sufficiency in growth signals, insensitivity to anti-growth signals, evading apoptosis, unlimited replication potential, and sustained angiogenesis, as well as tissue invasion and metastasis (25). Most review articles analyzed NSCLC from epidemiological perspectives, including, epidemiology, risk factors, treatments, and survival. The 7^th^ most frequently cited article discussed pembrolizumab, which is a humanized monoclonal antibody targeting the programmed death 1 (PD-1) protein. Pembrolizumab is associated with significantly longer progression-free and overall survival (OS) rates, has fewer adverse events when compared with platinum-based chemotherapy in patients with advanced NSCLC, and targets PD-L1 expression on at least 50% of tumor cells (26). The 9^th^ most frequently cited article reported that nivolumab had longer OS times when compared with docetaxel in patients with advanced non-squamous NSCLC, which had progressed during or after platinum-based chemotherapy (27).

**Table 5:** Top ten most cited references related to non-small cell lung cancer and metabolism.

Rank	Citations	Title	Author	Journal	Year	DOI
1	190	Hallmarks of cancer: the next generation	Douglas Hanahan	CELL	2011	10.1016/J.CELL.2011.02.013
2	180	Cancer Statistics, 2021	Rebecca L Siegel	CA-CANCER J CLIN	2021	10.3322/CAAC.21654
3	168	Global cancer statistics	Ahmedin Jemal	CA-CANCER J CLIN	2011	10.3322/CAAC.20107
4	141	Understanding the Warburg effect: the metabolic requirements of cell proliferation	Matthew G Vander Heiden	SCIENCE	2009	10.1126/SCIENCE.1160809
5	104	The biology and management of non-small cell lung cancer	Roy S Herbst	NATURE	2018	10.1038/NATURE25183
6	97	New response evaluation criteria in solid tumors: revised RECIST guideline (version 1.1)	E A Eisenhauer	EUR J CANCER	2009	10.1016/J.EJCA.2008.10.026
7	96	From RECIST to PERCIST: Evolving Considerations for PET response criteria in solid tumors	Richard L Wahl	J NUCL MED	2009	10.2967/JNUMED.108.057307
8	91	Pembrolizumab versus Chemotherapy for PD-L1-Positive Non-Small-Cell Lung Cancer	Martin Reck	NEW ENGL J MED	2016	10.1056/NEJMOA1606774
9	88	Nivolumab versus Docetaxel in Advanced Nonsquamous Non-Small-Cell Lung Cancer	Hossein Borghaei	NEW ENGL J MED	2015	10.1056/NEJMOA1507643
10	81	On the origin of cancer cells	Otto Warburg	SCIENCE	1956	10.1126/SCIENCE.123.3191.309

**Figure 6:**
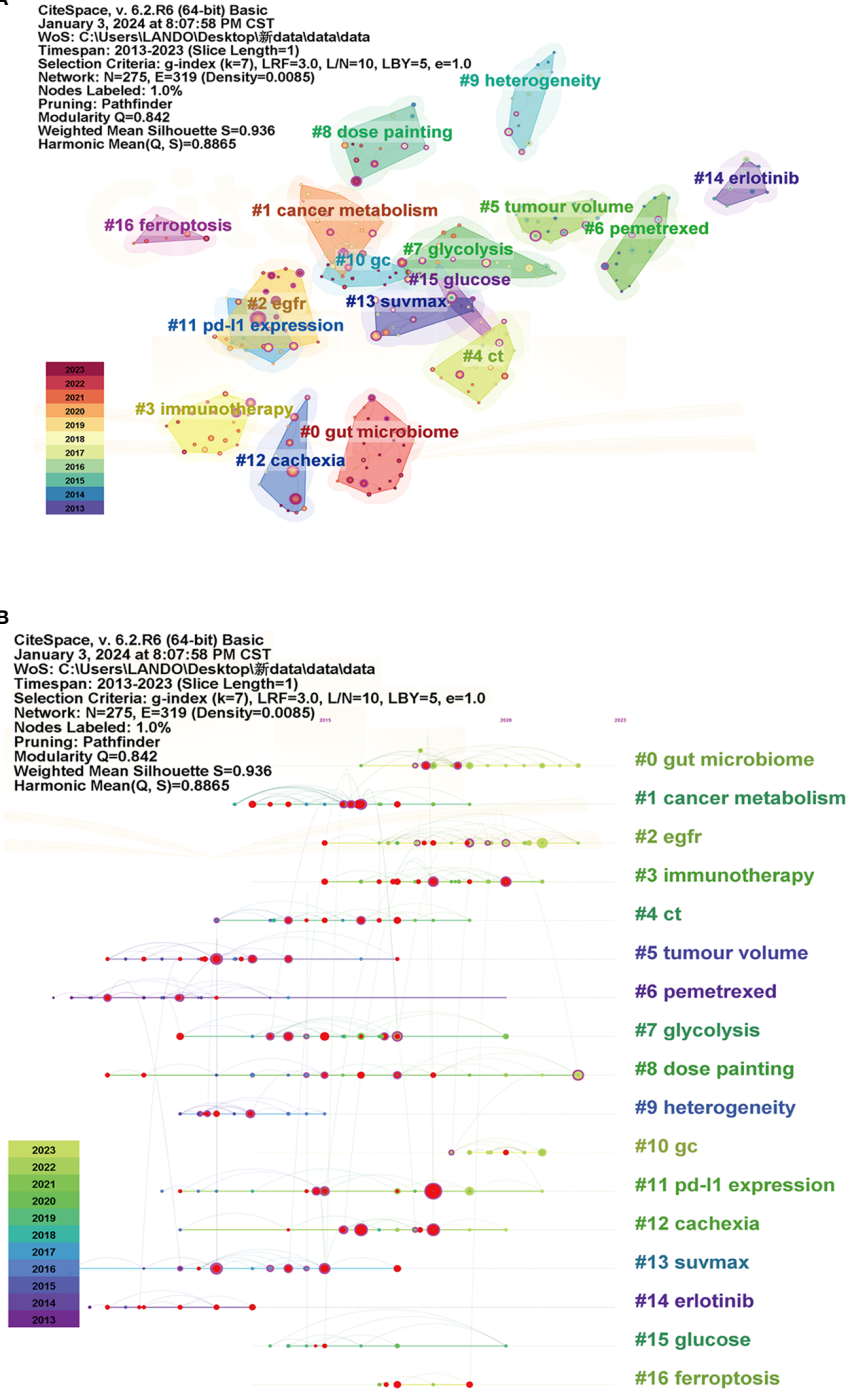
Cited reference analyses related to non-small cell lung cancer and metabolism (NSCLC-Met). **(A)** Reference cluster analyses. Each cluster represents a research topic, with its influence and relevance indicated by the size of the nodes and the connections. The larger the nodes, the more frequent the research activities related to the topic. **(B)** Cluster timeline distribution. Each cluster is labeled according to the year it first appeared, and different research topics are represented by lines of different colors.

We next plotted seven clusters which had the highest K-values (i.e., 8). Q and S cluster values were 0.842 and 0.936, respectively, and indicated that our analyses were of excellent quality, and included 17 clusters (**Figure 6A**). Based on the clusters, we further conducted a timeline (**Figure 6B**). The timeline of clusters showed that “gut microbiome”, “egfr” and “dose painting” were the relatively new selection and hotspots in the NSCLC-Met field.

**Figure 7:**
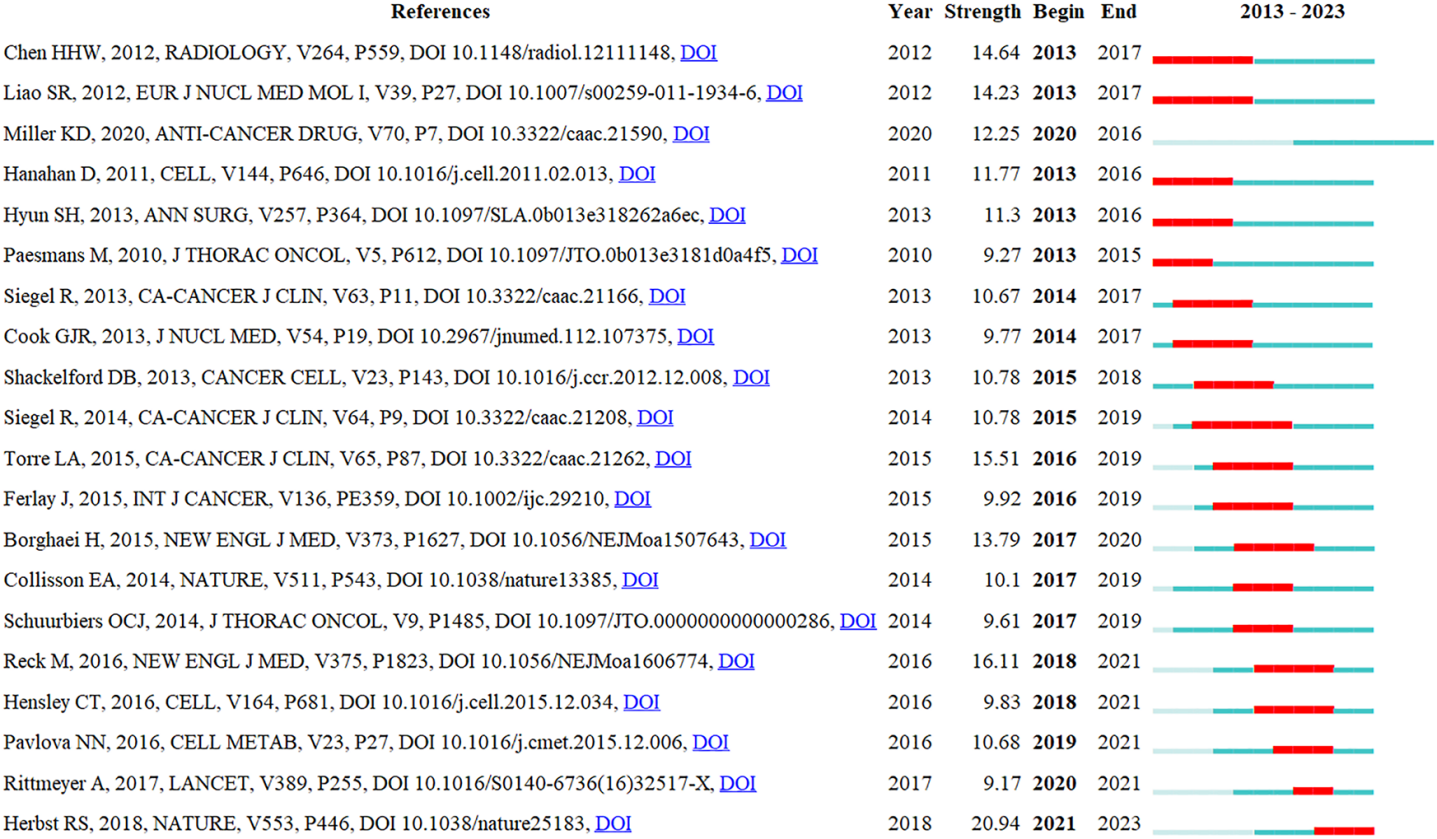
Top 20 references with the strongest citation bursts related to non-small cell lung cancer and metabolism (NSCLC-Met). Each reference’s burstiness is represented by the length of the line and the saturation of its color. Long and deeply colored lines indicate references with high burstiness, signifying a sharp increase in research interest in these references over a short period of time.

A reference burst analysis was then performed on the top 20 most strongly cited references (**Figure 7**). The article by Roy S Herbst (2018) had the highest burst strength (20.94), which was confirmed as a relative classic in recent years. This author summarized significant NSCLC treatment advances in the past 20 years, and showed that small molecule tyrosine kinase inhibitors and immunotherapy approaches in selected patients had generated unprecedented survival benefits in patients. However, a disease cure and NSCLC-survival rates remain low, especially for metastatic disease. Therefore, new drug and combination therapy research must continue to expand clinical benefits in more broader patient cohorts and improve NSCLC prognostics (28).

**Figure 8:**
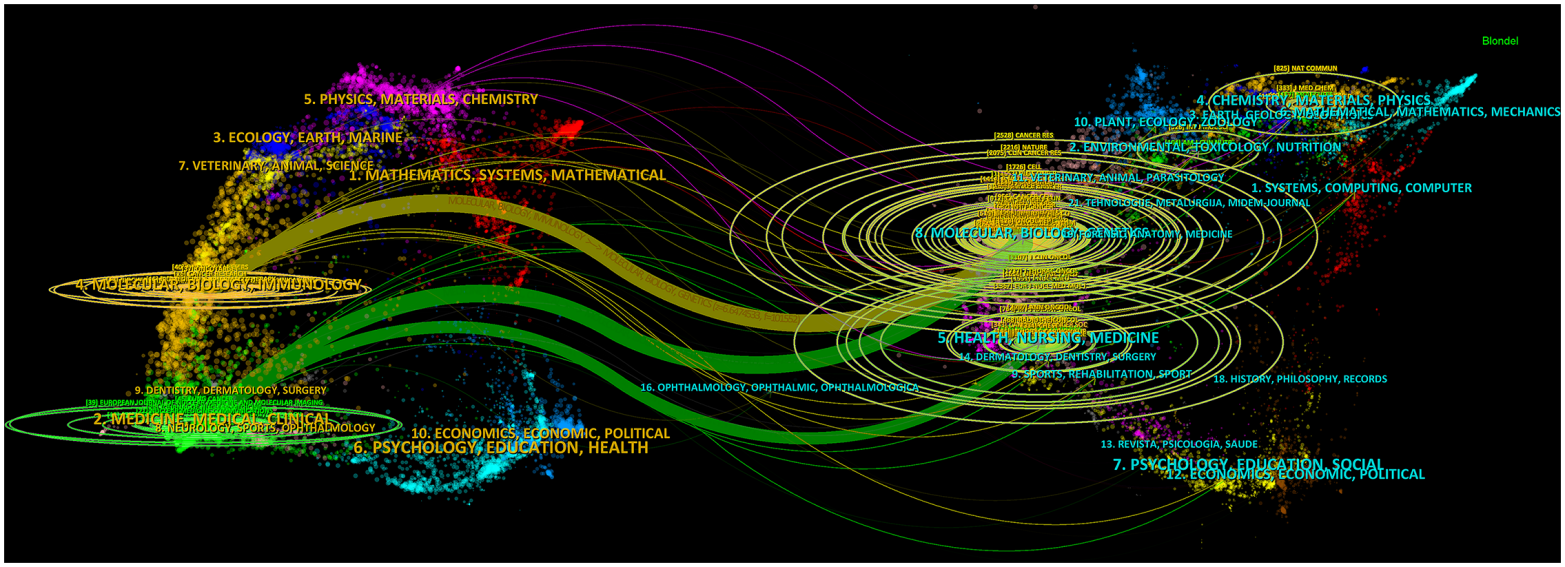
Dual-map overlay showing the disciplinary distribution of non-small cell lung cancer and metabolism (NSCLC-Met) articles. The articles and cited results were visualized. Wider edges indicated higher value in occurrence. The left part of the plot indicated citing journals, the right part of the plot indicated cited journals, and the curves represent citation relationships.

Finally, to visualize citation patterns, an overlay map was generated (CiteSpace) for double-map superposition analysis. The disciplinary distribution of NSCLC-Met articles is shown (**Figure 8**). The double-map overlay showed academic–discipline distributions and citation relationships of these citations; on the left, citing journals, and on the right, cited journals. Curves served as citation links to present reference origins and context. In the map (left side), the ellipse represented article numbers corresponding to journals and showed the ratio of authors to article numbers. Ellipse length represented the number of authors, and ellipse width represented article numbers (the more authors, the longer the horizontal axis of the ellipse, and the more articles a journal published, the longer the vertical axis of the ellipse). Curves between left and right map portions represented citation links, and their trajectories reflected interdisciplinary relationships in the field. Z-scores reflected stronger and smoother trajectories, with higher scores indicated by thicker connecting lines. As shown, molecular/biology/immunology citations (orange trajectory) were clearly influenced by articles in the molecular/biology/genetics field (z = 6.647 and f = 201552), and citations in the medicine/medical/clinical field (green trajectory) were influenced by articles in the molecular/biology/genetics field (z = 3.24 and f = 6239).

## Discussion

Globally, LC is one of the most deadliest malignant cancers (29). In recent years, novel research findings have uncovered a complex network of alterations in LC metabolism, including altered lipid-related pathways and biological functions. Consequently, regulatory mechanisms underpinning lipid metabolism have attracted considerable research attention in NSCLC etiology (11). Here, our bibliometric approach, incorporating CiteSpace, VOSviewer, and Bibliometrix, was used to investigate NSCLC-Met, visually represent current research, identify putative future hotspots and trends, and generate a comprehensive macroscopic overview of the NSCLC-Met field.

We identified 2,246 NSCLC-Met articles from WoSCC. A bibliometric analysis of the last decade indicated that annual article numbers had been increasing year on year since 2013. The entire research field showed a stable growth trend, was currently booming, and showed great developmental potential. It was noteworthy that in 2022, articles in this field numbered 352, which showed that scientists had maintained their high interest in the NSCLC-Met field in the preceding ten years.

*Cancers*, *Frontiers in Oncology*, and *PLoS ONE* were the top three most productive journals in the NSCLC-Met field. By analyzing the distribution of core journals, we provided guidance on manuscript submission and journal selection. In terms of citation frequencies, *Cancer Research*, *Nature*, and *Journal of Clinical Oncology* were the top journals, with another seven recognized as top journals. These journal analyses will undoubtedly help scientists identify important, recently published articles which reflect major research developments.

Across 66 countries, China was the most productive country. Based on article numbers and citation frequencies of different countries in relevant fields, we assessed their influence and research capacity. In terms of national article numbers, countries such as China and the USA were outstanding in the NSCLC-Met field, with highly impressive research outputs. Collaborations also occurred between countries, with China a central player in the field and several successful scientific collaborations with other countries. However, China was the only developing country on the list; the rest were all developed. However, of the top ten institutions, six were Chinese, but the University of Texas System led in terms of citations and article numbers. Overall, developed countries dominated research in the NSCLC-Met field.

Using our keyword co-occurrence network to analyze high-frequency keywords, several main research directions and hotspots were identified in the NSCLC-Met field. Keyword co-occurrence reflects the simultaneous occurrence of keywords in articles in a particular field, which indicates particular internal relationships. The more they appear, the stronger the correlation (30). Keyword co-occurrence networks can detect research structures and provide a dynamic analysis of disciplines; thus “expression” and “metabolism” keywords are most closely connected to surrounding keywords.

In keyword clustering analyses (CiteSpace), modularity Q and mean silhouette S metrics are based on network structures and clustering clarity, and may be used by scientists to assess cluster reasonableness (31). Typically, a modularity Q value > 0.3 suggests that delineated community structures are significant, while a mean silhouette S > 0.5 value reflects reasonable clustering. From keyword clustering parameters, the Q value was 0.7881 and the mean S value was 0.9067, which indicated a reliable analysis. Metabolic tumor volume represents tumor tissues with high metabolic activity; it is a strong prognostic and predictive factor in NSCLC patients receiving PD-1 inhibitor treatment, and is determined using routine ^18^F-FDG-PET/CT scans. Potentially used as a personalized immunotherapy, the approach may use to stratify patients in clinical studies (32). Positron emission tomography (PET) is a non-invasive imaging method typically used in medical diagnostics/scientific research; it can significantly improve NSCLC management and may improve prognosis outcomes and reduce the frequency of acute and late treatment-related adverse events (33).

In the NSCLC-Met field, in recent year, major outbreaks were identified for “lung adenocarcinoma”, “immunotherapy”, “nivolumab”, “checkpoint inhibitors”, “blockade”, and “pembrolizumab”, while the keyword with the longest outbreak duration was “gefitinib”. Therefore, it was possible that the NSCLC-Met field may be intensively studied in these areas in the coming several years. Article numbers for the immunotherapy-related keyword “nivolumab” were relatively low, as the term only exploded in 2020 and was not widely cited as an emerging treatment option. On October 9^th^ 2015, the US Food and Drug Administration accelerated the review and approval of nivolumab due to its significant benefits when compared with docetaxel in metastatic non-squamous NSCLC patients with respect to improved OS times and a combination-immunotherapy trial which attracted significant attention (34). The “pembrolizumab” keyword exploded as recently as 2021 and continues to this day. Immunotherapy has rapidly evolved in the recent 5 years. Pembrolizumab-combination chemotherapy was approved in 2017 and has brought new hope to the patient cohort without targeted mutations. As a first-line treatment for advanced non-squamous NSCLC, it provides patients with the option of an appropriate and personalized combination therapy (35). Several newly identified NSCLC targets, such as liver kinase B1 (LKB1) and kelch-like ECH-associated protein 1 (KEAP1), have attracted increasing attention in recent years (36). LKB1 and KEAP1 are strictly related to cell metabolism, and LKB1 or KEAP1 mutations are related to poor response to immunotherapy. Therefore, LKB1, KEAP, or the new hypothetic NSCLC targets may be found as outbreak keywords in the NSCLC-Met field in the coming years. Also, from the outbreak timeline (CiteSpace), current NSCLC treatment–research directions appeared to be shifting away from chemotherapy approaches to targeted therapies using biomolecules.

New NSCLC treatments are composed of tyrosine kinase inhibitors (TKIs) and immune checkpoint inhibitors (ICIs). The use of ICIs, monoclonal antibodies targeting PD-1, PD-L1 and cytotoxic T lymphocyte antigen 4 (CTLA-4), has yielded impressive results and is currently a cornerstone in the treatment of various cancers, including NSCLC. However, despite their availability, NSCLC survival rates remain low. The metabolic alterations that modify the tumor microenvironment (TME) play a central role in the development of resistance to ICIs (37, 38). Dysregulation of metabolic pathways, including the adenosine pathway and PI3K/AKT/mTOR pathway, affects T cell recruitment and metabolic activities in the TME, resulting in T cell exhaustion and anergy, impairing the immune control of tumor growth and the response to ICIs (39, 40). Several drugs targeting CD73, one of the ectonucleotidases that generate adenosine, have been proven efficacious in preclinical studies and mouse tumor models (41). The PI3K/AKT/mTOR pathway is physiologically involved in cell growth and survival, metabolism and motility, and its dysregulation in cancers results in tumor initiation, progression and drug resistance. Recent studies showed that an alteration of the PI3K/AKT/mTOR pathway might promote an immunosuppressive response, and PI3K inhibition induces a reduction in PD-L1 expression in tumor cells (42, 43). To address this, new drugs and specific measures are required, therefore, metabolic pathways implicated in NSCLC, as potential new disease targets, must be explored to ameliorate outcomes for NSCLC patients.

Glucose metabolic disorder is a tumor hallmark, including NSCLC, and inhibited tumor glycolysis directly hinders tumor cell survival and growth (44). The metabolism of NSCLC cells is heterogeneous. In addition to glycolysis and glucose oxidation, oxidation of non-glucose nutrients, such as lactate, also emerged as a potential carbon source (45). Aberrant lipid metabolism is observed in cancer. Several studies have reported that potent anti-tumor effects were exerted by inhibiting key lipid metabolism-associated enzyme activity in LC, such as fatty acid synthase (FASN) (46–48), stearoyl-CoA desaturase 1 (SCD1) (49–51), ATP citrate lyase (ACLY) (52), and statins (53). Overexpression of CD36, the scavenger receptor involved in lipid uptake, has been observed in a variety of tumors to protect cancer cells from treatment (54–56). Moreover, inhibition of CD36 could re-sensitize drug-resistant cells to immunotherapy and chemotherapy (54, 57). Tumor-associated macrophages (TAMs) are the most abundant immune cells in TME, and contain two different phenotypes, the classically (M1) and alternatively (M2) activated macrophages (58). M2 macrophages exhibit a tumor-promoting (immunosuppressive) effect, while M1 macrophages show a tumor-inhibiting effect (59). Several processes of lipid metabolism like fatty acid (FA) uptake, biosynthesis and storage are enhanced in TAMs (60). The immunosuppressive phenotype of TAMs is induced by unsaturated long-chain fatty acid metabolism (61). To obtain sufficient energy and ensure cancer cells survival, TAMs overexpress CD36 and accelerate FAs transport into cell for storage and oxidation, the enhanced fatty acid oxidation (FAO) leads to high rates of oxidative phosphorylation and STAT6 signaling pathways, which ultimately regulate gene transcription to determine the function of TAMs (62). Moreover, inhibition of CD36 may be a potential strategy for immunotherapy. Therefore, further studies examining relationships between metabolism and LC, and identifying potential disease therapeutic targets, may provide new clinical treatments for LC.

Our study had some limitations. Firstly, despite the wide range and coverage of selected search terms, it was possible that some articles were missed. Secondly, we only analyzed the literature from 2013 to 2023, possibly failing to include a few significant studies before this period. This may have caused us to overlook key research that defined early developments in the field, potentially affecting our understanding of the overall progress in the area. Additionally, we only used the WoSCC database and did not include articles from other databases, such as PubMed or Scopus, which may have caused a selection bias in our analyses. Similarly, our bibliometric conclusions may differ from the actual research situation, e.g., some recent and high-quality articles may have been overlooked due to low citation numbers. Despite these limitations, we believe our study highlights future trends and general developments in this important subject area. Future research should consider expanding the range of literature and databases to enhance the comprehensiveness and accuracy of the research.

## Conclusions

Our study is the first comprehensive visualization and bibliometric analysis of the NSCLC-Met field. The annual article output (numbers) in this field showed a continuous upward trend and rapid development over the last ten years, suggesting extensive and considerable interest by the global research community. From our study data, scientists can now explore recent hotspots and new research avenues in the NSCLC-Met field, and may benefit from our target journal data in terms of publishing articles. Among the major outbreak keywords identified in recent years, the immunotherapy will become a new research avenue for the NSCLC-Met field, in addition to therapeutic drugs and imaging methods. Further in-depth research on the NSCLC-Met field will undoubtedly provide new insights for disease diagnosis, treatment, and prognosis outcomes.

## Data availability statement

Original contributions are included in the article. Further inquiries may be directed to the corresponding authors.

## Author contributions

JY: Writing – original draft, Writing – review & editing. WY: Conceptualization, Writing – original draft. JZ: Resources, Writing – original draft. AH: Software, Writing – original draft. SY: Resources, Writing – original draft. HZ: Methodology, Writing – original draft. ZL: Data curation, Writing – original draft. XL: Resources, Writing – original draft. YC: Writing – review & editing. LM: Writing – review & editing. CW: Writing – review & editing.
